# Rhabdomyolysis-Induced AKI (RIAKI) Including the Role of COVID-19

**DOI:** 10.3390/ijms23158215

**Published:** 2022-07-26

**Authors:** Ewelina Młynarska, Julia Krzemińska, Magdalena Wronka, Beata Franczyk, Jacek Rysz

**Affiliations:** Department of Nephrology, Hypertension and Family Medicine, Medical University of Lodz, ul. Żeromskiego 113, 90-549 Lodz, Poland; julia.krzeminska1@gmail.com (J.K.); wronkam96@gmail.com (M.W.); bfranczyk-skora@wp.pl (B.F.); jacek.rysz@umed.lodz.pl (J.R.)

**Keywords:** rhabdomyolysis, acute kidney injury, statin, COVID-19, renal replacement therapy

## Abstract

Rhabdomyolysis is a compound disease that may be induced by many factors, both congenital and acquired. Statin therapy is considered one of the most common acquired factors. However, recent scientific reports suggest that serious complications such as rhabdomyolysis are rarely observed. Researchers suggest that, in many cases, side effects that occur with statin therapy, including muscle pain, can be avoided with lower-dose statin therapy or in combination therapy with other drugs. One of the most recent agents discovered to contribute to rhabdomyolysis is COVID-19 disease caused by severe acute respiratory syndrome coronavirus 2 (SARS-CoV-2). Rhabdomyolysis is defined as a damage to striated muscle cells with escape of intracellular substances into the bloodstream. These substances, including myoglobin, creatine kinase (CK), potassium, and uridine acid, are markers of muscle damage and early complications of rhabdomyolysis. Symptoms may be helpful in establishing the diagnosis. However, in almost 50% of patients, they do not occur. Therefore, the diagnosis is confirmed by serum CK levels five times higher than the upper limit of normal. One of the late complications of this condition is acute kidney injury (AKI), which is immediately life-threatening and has a high mortality rate among patients. Therefore, the prompt detection and treatment of rhabdomyolysis is important. Markers of muscle damage, such as CK, lactate dehydrogenase (LDH), myoglobin, troponins, and aspartate aminotransferase (AST), are important in diagnosis. Treatment of rhabdomyolysis is mainly based on early, aggressive fluid resuscitation. However, therapeutic interventions, such as urinary alkalinization with sodium bicarbonate or the administration of mannitol or furosemide, have not proven to be beneficial. In some patients who develop AKI in the course of rhabdomyolysis, renal replacement therapy (RRT) is required.

## 1. Introduction

Rhabdomyolysis is potentially a life-threatening group of symptoms that develops as a result of muscle cell destruction and the release of intracellular components into the bloodstream [1,2]. It is characterized by an acute or subacute onset with associated muscle pain, muscle weakness, and dark colored urine [1,3]. This is the classic triad of symptoms. However, it occurs in only about 10% of patients, while up to 50% of patients present no clinical symptoms [3]. Rhabdomyolysis can be triggered by many causes, among which we can mention congenital causes like muscular dystrophies and acquired causes, such as trauma, infections, and some drugs [4]. The most important medications that can cause skeletal muscle-associated symptoms (SAMS), including the most severe form, rhabdomyolysis, are statins [5,6]. Statins are selective inhibitors of HMG-CoA reductase, which reduce blood cholesterol levels [6,7]. It is estimated that 5–10% of patients experience muscle pain during therapy [8], whereas the occurrence of serious side effects of statins such as muscle damage is rare [6]. Researchers suggest that the use of lower doses of statins, for example in combination with other low-density lipoprotein (LDL)-cholesterol-lowering drugs, reduces the risk of developing serious myopathies. They also recommend such a therapeutic model for patients in whom high-dose statin monotherapy caused rhabdomyolysis [9,10].

COVID-19 is a disease caused by severe acute respiratory syndrome coronavirus 2 (SARS-CoV-2) [11], which has many variants that change the characteristics of the disease affecting, among other things, the method of transmission or treatment. The disease continues to spread worldwide causing new infections and leading to deaths [12]. SARS-CoV-2 spreads rapidly by respiratory droplets and direct contact. Although the disease can be characterized by a variety of symptoms, the most common include fever, cough, fatigue, sputum production, shortness of breath, as well as myalgia and arthralgia [11]. COVID-19 is also among the causative factors of rhabdomyolysis [13,14,15,16,17]. Some investigators have implicated excessive immune response in the causes of muscle damage during SARS-CoV-2 infection [13,18]. Others point to direct damage caused by the virus or involving immune factors [19]. Acute kidney injury (AKI) is also a common complication of COVID-19 [20,21,22]. Many causes of its onset have been cited, one of which is rhabdomyolysis leading to intratubular obstruction [23].

Regardless of the cause, damage to muscle cells can have significant consequences, leading to the development of complications. The most serious complication is AKI, which develops in up to 7–10% of patients and has a high mortality rate of 30–50% among hospitalized patients [24,25,26]. For this reason, it is important to detect and commence the treatment of rhabdomyolysis promptly [27].

## 2. Basic Information about Acute Kidney Injury (AKI)

AKI, until recently described as acute renal failure, is a collection of various kidney injuries, including renal failure [24,28]. It is characterized by a sudden deterioration of renal function, manifested as a rapid decrease in glomerular filtration rate (GFR) within hours/days, and is usually reversible [24,29]. There are several criteria for the diagnosis of acute renal failure, the most common of which are the KDIGO criteria. According to these criteria, confirmation of an increase in serum creatinine concentration or a decrease in urine output volume is sufficient for the diagnosis of AKI [24,25,29,30]. Table 1 shows the components of KDIGO. One criterion is sufficient to diagnose AKI [24,25,30].

AKI may be caused by many different causes and we divide them into three categories due to their pathophysiology: prerenal, intrarenal, and postrenal. Prerenal are caused by a reduction in blood flow through the kidneys, and among them we can mention hypotension, reduction in the volume of circulating blood or the use of angiotensin converting enzyme inhibitors (ACEI). Intrarenal causes include parenchymal diseases, such as acute tubular necrosis (e.g., through the use of nephrotoxic agents), acute interstitial nephritis, and rhabdomyolysis. Postrenal causes are due to a disturbance in urine outflow e.g., obstruction of the urinary tract by stones or external pressure [24,25,29]. Table 2 contains three categories of AKI causes with examples [24,25,29].

The laboratory and imaging tests used in the diagnosis make it possible to determine the etiology of AKI. In prerenal AKI, urine analysis (UA) reveals vitreous or granular casts but can also be normal. In the measurement of serum creatinine, its concentration does not rise as rapidly as in acute tubular necrosis injury, while in the urine osmolality test, values are above 500 mOsm/kg. In the fractional excretion of sodium (FENa) study, values of less than 1% are characteristic of pre-renal AKI, and urine sodium concentration is less than 20 mEq/L [31,32]. In acute renal tubular necrosis, examination of the urine sediment reveals brown deposits, and the serum creatinine concentration is characterized by a rapid rise. The urine osmolality test presents reduced values at less than 450 mOsm/kg due to impaired urine concentration, while in FENa study, acute renal tubular necrosis takes values above 2% with urine sodium concentration reaching values above 40% [24,31,32]. Among the renal causes of kidney damage, some of them have several characteristics. In interstitial nephritis, we can see deposits of white blood cells in the urine sediment, sterile pus, and proteinuria, while microscopic hematuria is also characteristic [24,33]. UA of glomerulonephritis can detect dysmorphic erythrocytes as well as microscopic hematuria or proteinuria [34]. Rhabdomyolysis is also one of the causes of renal AKI distinguished by features in laboratory tests. In the course of rhabdomyolysis-induced AKI (RIAKI), a brown coloration of the urine and brown urine supernatant are observed due to the excretion of myoglobin, in addition, there are significantly elevated serum creatinine kinase values. A high serum myoglobin concentration is also characteristic, but due to its unpredictable metabolism, its measurement has low sensitivity. Other indicators of RIAKI may include a low blood urea nitrogen to creatinine ratio, oliguria, low fractional sodium excretion and electrolyte abnormalities, which include metabolic acidosis with a large anion gap, hyperkalemia or hipercalcemia [35]. Obstruction of urine outflow by urolithiasis, among others, also contributes to the development of kidney damage, and imaging studies, such as ultrasound or computed tomography without contrast, are applicable for its detection, while leukocytes, nitrites, blood, or microscopic hematuria can be detected in UA [25,36].

AKI may develop many complications, the most common of which are metabolic disorders, such as hyperkalemia, metabolic acidosis, hyperphosphatemia, and peripheral edema. Moreover, complications may involve other organs and organ systems, including the gastrointestinal tract, central nervous system, and cardiovascular system, contributing to heart failure, arrhythmias, or myocardial infarction [24].

Acute kidney damage can resolve completely with the treatment in most cases. However, a certain percentage of patients are associated with increased mortality. There is a variety of different factors related to AKI prognosis, the most significant of which are severity and duration of kidney damage. Patients with a low estimated glomerular filtration rate (eGFR) that persists for a long time are more likely to develop end-stage renal failure, thus contributing to increased mortality in this group of patients [24]. Among hospitalized patients, the most common cause of AKI is acute tubular necrosis, which can cause irreversible damage and has a high patient mortality rate [24,31]. A more severe course of the disease, as well as increased mortality, also affects patients who are elderly, have hypotension, need transfusions, oliguria, or multi-organ dysfunction [8].

## 3. AKI as a Complication of Rhabdomyolysis

**Figure 1:**
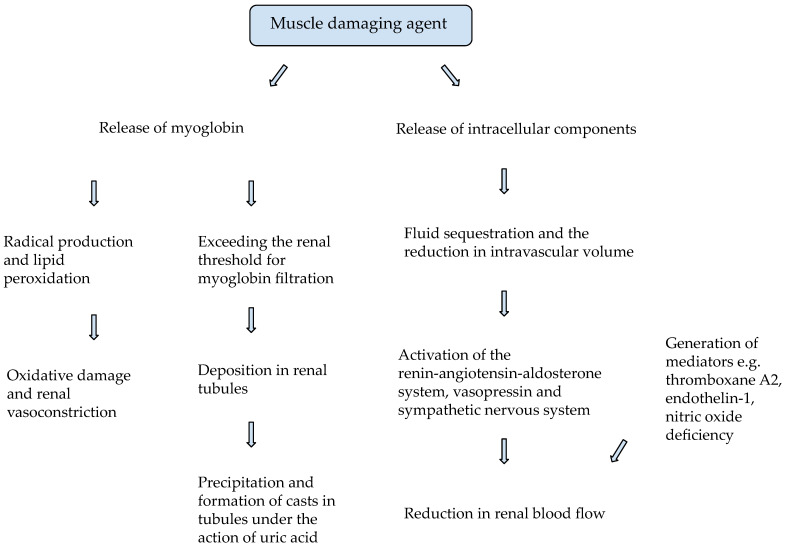
Pathomechanism of rhabdomyolysis-induced AKI (RIAKI) [2,26,27,35,37,38,39,40].

Rhabdomyolysis may contribute to the development of AKI in up to 7–10% of patients, and the mechanism of this process is compound [26]. After the muscle-damaging agent has acted, its intracellular contents are released into the bloodstream, causing fluid sequestration and subsequent reduction in intravascular volume. This process results in activation of the renin-angiotensin-aldosterone system, leading to a decrease in renal blood flow through renal vasoconstriction [2,26,37]. A reduction in renal blood flow is also compounded by activation of vasopressin, the sympathetic nervous system and generation of mediators e.g., thromboxane A2, endothelin-1, and nitric oxide deficiency [35]. Myoglobin released from damaged muscles is a major renal injury factor deposited in renal tubules [38]. Under physiological conditions, myoglobin undergoes natural filtration in the glomeruli. However, during muscle breakdown, excessive amounts of myoglobin are released, exceeding the renal threshold, leading to myoglobinuria and renal damage [26,38]. As an iron-containing protein, it has the ability to bind molecular oxygen, which may produce a hydroxyl radical in the oxidation of ferrous oxide (Fe^2+^) to ferric oxide (Fe^3+^) [35]. Nephrotoxic effects of myoglobin through free radical production and lipid peroxidation leading to renal vasoconstriction and oxidative damage to renal tubules also contribute to the development of AKI [2,38,39]. Metabolic acidosis and increased uric acid concentrations potentiate the nephrotoxic properties of myoglobin through its precipitation and interaction with Tamm-Horsfall protein to form casts in tubules [2,26,27,38,40]. Rhabdomyolysis also causes an increase in uric acid, which under acidic conditions is mainly deposited at the level of distal tubules in the form of crystals, impeding urine flow [2]. Summing up, renal function in RIAKI is impaired mainly due to the nephrotoxic effects of myoglobin on renal tubule cells, the deposition of casts in renal tubules with their subsequent obstruction, and renal vasoconstriction [41]. Figure 1 shows a diagram illustrating the pathomechanism of RIAKI [2,26,27,35,37,38,39,40]. The McMahon index is a specific score that is used to assess the risk of developing renal failure requiring renal replacement therapy (RRT) in patients with rhabdomyolysis on admission. This score takes into account gender, age, etiology, creatine kinase (CK), and other laboratory values, such as acidosis and hyperphosphatemia [26,42]. The use of the McMahone score may be a valuable tool in predicting the necessity for intensive treatment of patients without the need to wait for high CK values in the laboratory test [26,42]. Cabral B. et al. [26] and Simpson J. et al. [42] showed that a score of 6 or more had a higher specificity and sensitivity than an increase in CK levels >5000 U/L in predicting the occurrence of AKI or the requirement for dialysis. Correspondingly, a score of 5 or less was associated with a lower risk of developing AKI and the need for RRT [26,42]. Simpson et al. [42] suggest that having a high McMahone score on admission, rather than a CK value, may be helpful in identifying patients at high risk of kidney damage. On the other hand, Vangstad M. et al. [43] attempted to determine the relationship between myoglobin, CK, and the risk of developing AKI based on serum creatinine concentrations. It has been demonstrated that the use of the myoglobin/CK ratio in the diagnosis of patients with rhabdomyolysis can be a useful tool considering any suspicion of AKI, as a high ratio may suggest a high risk of developing this complication [43].

## 4. Rhabdomyolysis—Basic Information and Diagnostic Pathway

Rhabdomyolysis is a state of skeletal muscle destruction as a result of the breakdown and necrosis of striated muscle tissue. In this process, a significant number of intracellular enzymes enter the bloodstream, ultimately leading to various systemic complications [2,4,26,44].

Despite the wide spectrum of causes of rhabdomyolysis, the mechanism of rhabdomyolysis is the same in each case and comes down to an increase in the amount of ionized intracellular calcium. As a result of damage to the cell membranes of muscle cells, transmembrane proteins are destroyed, including ion channels and pumps, the most important of which are the Na-K-ATP pump and the Na^+^/Ca^2+^ exchanger, which allow the cell to maintain a negative ionic potential and low calcium concentration [2,26]. The depletion of cellular ATP reserves also impairs the proper functioning of pumps and ion channels also leading to an uncontrolled increase in calcium concentration in myocytes [35]. As a consequence, calcium homeostasis is disturbed and excessive amounts of ionized calcium accumulate in the cells, which activates proteolytic enzymes, ultimately leading to apoptosis and the release of large amounts of CK, potassium, and myoglobin into the bloodstream [2,26].

After exposure to the causative agent, symptoms of rhabdomyolysis develop within hours/days in the form of a classic triad of symptoms including brown urine pigmentation, muscle pain and weakness [1,2,3,4,26]. However, the above-mentioned symptoms occur in less than 10% of patients [3]. In addition, patients may present swelling and muscle cramps, scant urination, and a number of other nonspecific symptoms, including dysrhythmias due to dyselectrolytemia [1,2,3,4,26]. Importantly, nearly 50% of patients may present no symptoms [3].

There are many methods for diagnosing rhabdomyolysis. Laboratory tests play a key role in the diagnosis, among them serum CK measurements or urine myoglobin determinations are the most common. Rhabdomyolysis is characterized by a gradual increase in CK concentration, reaching its peak 24–72 h after the triggering factor. Measurement of CK concentration appears to be the most reliable biochemical marker of skeletal muscle damage, with the CK-MM subtype having the highest sensitivity and its fivefold increase above the upper limit of normal being the gold standard for diagnosis. Myoglobinuria results from the rapid renal excretion of myoglobin released from damaged muscle, and a visible change in urine color occurs at values >100 mg/dL of myoglobin in urine [4,26]. UA, which can detect hematuria and proteinuria, may be a helpful tool in making the diagnosis. Electrolyte testing also provides us with a lot of relevant information, in which hyperkalemia, hyperphosphatemia, hypercalcemia or hypocalcemia, increased creatinine and bilirubin, increased anion gap and hyperuricemia may be present [26]. In addition to the methods mentioned above, there are other methods that are less common but allow for the differential diagnosis of acquired causes from genetic causes of rhabdomyolysis. Among these methods, we can mention the forearm exercise test (FET), acylcarnitine profile, graded exercise testing, muscle biopsy, and genetic testing [4].

### 4.1. Muscle Injury Markers

As mentioned before, rhabdomyolysis, besides clinical manifestations, is also manifested in laboratory parameters [4,26]. Muscle damage can be demonstrated by elevation of parameters such as CK, lactate dehydrogenase (LDH), myoglobin, troponins, and aspartate aminotransferase (AST) [45].

CK is a marker of myocytes damage. Its elevated serum levels coexist with muscle disorders [46], as well as an increase in CK serum levels can be observed after physical activity [47]. The CK value is a parameter that is not only the basis for the diagnosis of rhabdomyolysis, but also an indicator of progression to AKI [45]. We distinguish CK-MM, CK-MB, and CK-BB occurring in the cytoplasm and sarcomeric and non-sarcomeric isoenzymes occurring in mitochondria [48].

As well as CK, LDH level is a marker of muscle damage, so its level will be significantly elevated in patients with rhabdomyolysis. In addition, it has been observed that LDH levels are increased in conditions where cell necrosis occurs e.g., myocardial infarction or cancer. It has been proven that LDH is a sensitive marker for the onset of AKI as a complication of rhabdomyolysis [49].

Myoglobin is a hemoprotein that, among other things, stores and transports oxygen. Its detection in urine is indicative of myocardial infarction or muscle damage, and thus also of the risk of AKI [46,48].

Troponins are markers of not only cardiac but also skeletal muscle damage [46]. In skeletal muscle damage, in contrast to cardiomyocytes, there is an isolated increase in cardiac troponin T (cTnT) levels. However, it has been proven that the increase in troponins in the course of rhabdomyolysis is probably caused by the disease process responsible for rhabdomyolysis, which causes damage to cardiomyocytes [50].

AST is an enzyme with a wide distribution in the body; it is present in the liver, muscle, heart, and erythrocytes. Its clinical significance is mainly attributed to the diagnosis of liver disorders. However, elevated levels are also observed in muscle damage [48]. It has been shown that the enzyme strongly correlates with CK. Indeed, its value allows to predict CK concentrations. Therefore, when CK cannot be measured, AST determination will provide crucial information [45].

### 4.2. Causative Factors of Rhabdomyolysis

The breakdown of striated muscle can arise as a result of many different causes. Predisposition to the development of rhabdomyolysis can be divided using several classifications, including a very common division into genetic/acquired causes [2]. Genetic causes include muscular dystrophies, channelopathies, metabolic myopathies, mitochondrial disorders, disorders of glycolysis, glycogenolysis, and lipid metabolism. A characteristic feature of genetic defects resulting in metabolic disorders is recurrent episodes of rhabdomyolysis. Patients with structural myopathy are at risk of developing an acute incident of rhabdomyolysis during muscle-toxic pharmacotherapy, during anesthesia or while performing intense exercise [4,35,38]. Acquired causes include alcohol, drugs, some prescription medications, including antipsychotics, antidepressants, statins, and fibrates, electrolyte disturbances, and metabolic diseases in the course of many diseases, such as diabetes, thyroid dysfunction, or primary hyperaldosteronism, ingestion of poisons, pesticides and toxins, e.g., contained in some species of fungi, autoimmune myopathies as well as crush injuries [4,26,38,44,51]. Rhabdomyolysis resulting from traumatic causes like accidents or crushing develops only after the pressure is eliminated and the subsequent release of toxic substances from the damaged muscles [26]. Moreover, acquired causes include intense muscle activity during increased exertion, epileptic status or convulsions, exposure to very high temperatures in malignant neuroleptic syndrome, heat stroke or malignant hyperthermia, as well as prolonged immobilization during coma or surgery and numerous infections, e.g., viral or bacterial [2,4,26,38,44,51]. Different age groups have different predominant causes of rhabdomyolysis, and so viral infections, trauma and drugs are most common in children, while drugs and trauma predominate in adults [2].

Ayala E. et al. [51] presented a case report of a 28-year-old woman where rhabdomyolysis was associated with the development of influenza A (H1N1) viral pneumonia. The patient sought medical attention due to fever, dyspnoea, and muscle pain. Despite the antibiotic therapy, symptoms persisted. Laboratory tests on admission were notable for high LDH 1875 U/L and CK level 27,820 U/L. Because of the fear of the development of AKI due to rhabdomyolysis, the patient was given intravenous sodium bicarbonate and intensive fluid therapy, after which the high CK level decreased. It is worth noting that other potential causes of rhabdomyolysis were ruled out in history [51].

Fodili F. et al. [44] have also described a case of a healthy 32-year-old man who came to the hospital with muscle pain and brown-colored urine. For five days, the patient complained of high fever up to 40 °C, sore throat, and malaise. Other potential causes of rhabdomyolysis were ruled out in the history and a family history of musculoskeletal and renal diseases was excluded. Laboratory tests revealed a CK level of 250,000 U/L, LDH level of 14,160 U/L and serum creatinine on admission of up to 273 mol/L. The urine test revealed mild proteinuria, leukocyturia, and mild hematuria. Serology confirmed that the patient had a recent Coxackie B2 virus infection. After intensive fluid therapy, administration of sodium bicarbonate and hemodialysis, a decrease in CK levels and resolution of muscle pain were achieved within two weeks [44].

The cited case reports confirm that isolated viral infections may cause the development of rhabdomyolysis in patients. Table 3 shows some of the causes of rhabdomyolysis [2,4,26,35,38].

Statins are selective inhibitors of HMG-CoA reductase, thus inhibiting the conversion of HMG-CoA to mevalonate and then to cholesterol in liver cells [52]. In addition, statin-related reductions in circulating lipoprotein concentrations increase hepatic low-density lipoprotein receptor (LDLR) expression and clearance of circulating LDL, contributing to further reductions in blood cholesterol concentrations [6].

Potentially dangerous among the side effects of statins is myopathy, especially that leading to AKI or even death, rhabdomyolysis [6]. Although the authors emphasize that the mechanism of SAMS is unclear, the following theories are proposed, as shown in Table 4 [5].

Since it is known that as renal function deteriorates, the risk of side effects of medications taken by patients including statins, increases [53], maximum doses of statins have been established for patients with chronic kidney disease (CKD). Such a measure is aimed at avoiding side effects, among others, muscle-related [54].

A nationwide cohort study that included 8,236,667 people showed that the incidence and risk of rhabdomyolysis in people starting statin treatment for primary prevention of cardiovascular disease is low. However, the authors of the study emphasize the importance of starting treatment with lower doses, as well as careful patient monitoring [55]. This observation is also confirmed by the reports of the ESC/EAS guidelines on the management of dyslipidemias, which state that the occurrence of serious adverse effects of statins in the form of muscle damage is rare. Moreover, if muscle symptoms occur, it is usually possible to continue statin therapy, provided that the treatment is modified by reducing the dose or changing the statin used [6].

Moreover, the guidelines indicate that the therapy of patients at risk of atherosclerotic cardiovascular disease should be based on an individual choice of treatment depending on, among other things, clinical status, other medications used, or tolerance of the therapy by the patient. Importantly, it allows the use of additional drugs, such as PCSK9 inhibitors or ezetimibe in the form of combination therapy, noting that the best effect of PCSK9 inhibitors is obtained in co-administration with statins [6].

A study of 83,880 patients found that the vast majority of side effects attributed to statins-including muscle pain were equally common when patients took placebo [56]. The Anglo-Scandinavian Cardiac Outcomes Trial-Lipid-Lowering Arm study reached similar conclusions, finding that patients were significantly more likely to report muscle pain when they knew what drug they were taking. The researchers explain the results of the study by the negative attitude of the study participants towards statin therapy, the so-called nocebo effect [57].

### 4.3. Complications of Rhabdomyolysis

Intracellular components released into the bloodstream in the process of rhabdomyolysis contribute to the development of many complications. Depending on the time of onset, these can be divided into early, early or late and late complications. Early complications develop within 12 h and include hypocalcemia, hyperkalemia and hyperphosphatemia. Early or late complications include compartment syndrome developing within 12–24 h, while late complications include AKI developing more than 24 h after the damaging agent. The development of AKI poses the greatest risk to the patient [38].

Potassium is mainly stored inside the body’s cells, so their damage leads to hyperkalemia, which can cause life-threatening cardiac arrhythmias that can eventually contribute to cardiac arrest. Damaged muscle cells also release organic acids, which aggravate metabolic acidosis. Hyperphosphatemia is another early complication of rhabdomyolysis, resulting from the efflux of inorganic phosphorus from damaged cells into the bloodstream and impaired urinary phosphorus excretion [58]. In the early stages of rhabdomyolysis, calcium levels are reduced due to the precipitation of calcium phosphates and the accumulation of calcifications in damaged muscle fibers. However, over time, hypercalcemia begins to predominate, resulting from the release of intracellular calcium into the plasma [38,58].

**Figure 2:**
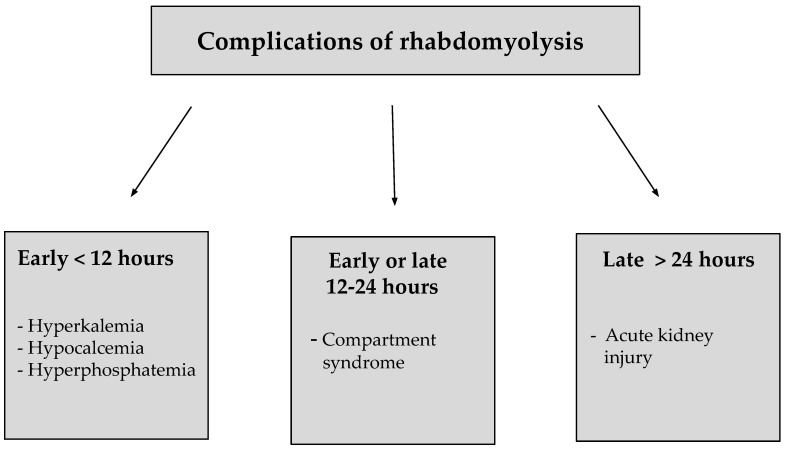
Classification of complications of rhabdomyolysis according to time criteria [38].

Compartment syndrome is caused by the influx of large amounts of calcium and sodium in damaged tissues, which entail significant amounts of extracellular fluid that contribute to the development of local edema. The resulting increased intramuscular pressure disturbs local blood flow and impedes venous blood return, exacerbating the edema. Compartment syndrome most commonly involves the biceps brachii, triceps brachii, wrist flexors and wrist extensors, as well as the thigh and gluteal compartments. In the course of this complication, patients experience symptoms, such as weak or absent pulse, local pain, and pallor, as well as motor and sensory disturbances if severe ischemia develops. AKI is one of the late complications of rhabdomyolysis and it has been discussed below [58]. Candela N. et al. [59] have demonstrated that a history of rhabdomyolysis complicated by the development of AKI may also lead to CKD. In this multicenter retrospective study, nearly one third of patients developed a significant decrease in eGFR greater than 20 mL/min/1.73 m^2^ at thre months, and some patients developed CKD [59]. Examples of complications of rhabdomyolysis are shown in Figure 2 [38].

## 5. SARS-CoV-2-Induced Rhabdomyolysis

**Figure 3:**
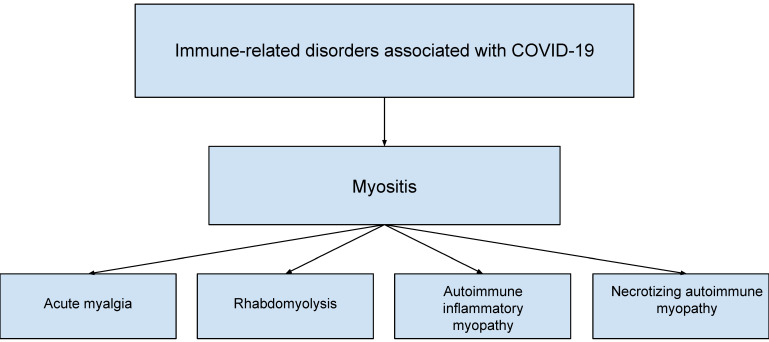
Immune-related disorders associated with COVID-19.

Despite the fact that COVID-19 manifests primarily with respiratory symptoms, it can also produce extrapulmonary symptoms [18]. One of these is myalgia, which is a common symptom of COVID-19, and is estimated to occur in 19–33% of patients [19]. Muscle symptoms include myositis, which is shown in Figure 3 [18]. As reported by Ramos-Casals et al. [18], myositis in COVID-19 occurred significantly more often in men than in women, among whom 33.3% required intensive care unit (ICU) hospitalization and 8.3% died. Myositis and/or rhabdomyolysis should be suspected especially in patients experiencing severe myalgia concurrent with CK levels >10,000 U/L.

Rhabdomyolysis is a rare complication of COVID-19 and occurs in approximately 0.2–2.2% of hospitalized patients. However, it can result in life-threatening consequences. In a report by Hannah et al. [19], slightly more than half of patients with rhabdomyolysis developed AKI. In addition, multiple organ failure or death are possible complications. Ramos-Casals et al. [18] point out that there are differences in the characteristics of clinical symptoms depending on age, gender or ethnicity. This is supported by the observations of Hannah et al. [19], who note that of patients with rhabdomyolysis in COVID-19, as many as 77% are male. They also point out that comorbidities, such as arterial hypertension, obesity, and diabetes mellitus, increase the risk of rhabdomyolysis in the course of COVID-19. Potential triggers of rhabdomyolysis in SARS-CoV-2-infected patients also include drugs, especially statins and antibiotics [19].

Some authors emphasize that the pathophysiological mechanism of rhabdomyolysis in COVID-19 is not clear [13,16,17]. Potential causes include direct muscle damage by the virus [13] or toxic effects on myocytes in the course of an excessive immune response [13,18]. Hannah et al. [19] report that SARS-CoV-2 enters cells via angiotensin-converting enzyme 2 (ACE2), which is expressed in skeletal muscle. However, they do not exclude a mechanism in which there is direct damage caused by the virus, or with the involvement of immune factors. There is cell damage caused by increased levels of interleukin-6 (IL-6). This results in metabolic acidosis and hyperlactatemia, making erythrocytes unable to carry oxygen, leading to hypoxemia. LDH activity and lactate production increase in the muscles, further exacerbating hypoxemia. ATP depletion and abnormal functioning of ion channels occur, ultimately leading to rhabdomyolysis, in the course of which muscle fibers are necrotic and metabolites are released into the bloodstream [19].

### 5.1. AKI in the Course of COVID-19 Including RIAKI

Several cases have been described in which coronavirus-induced rhabdomyolysis resulted in AKI [13,15,16,17]. However, there are instances in which this has been avoided [14]. Although the incidence of AKI in COVID-19 has shown a decreasing trend over time [60], it remains a common complication of SARS-CoV-2 infection [20,21,22]. AKI prolongs the duration of hospitalization and worsens the prognosis and increases the mortality of COVID-19 patients [21,23,61]. The factors that are associated with an increased risk of developing AKI have been identified and include older age [20,23], male gender [23,60], black race, and comorbidities such as diabetes mellitus [20,23,60], cardiovascular diseases, hypertension [20,23], CKD, or obesity [23,60].

Infection affects the kidney at the tubular, vascular, and glomerular levels [22]. AKI in COVID-19 can occur as a result of direct renal infection, as well as tubular and/or endothelial damage. Pigment casts resulting from rhabdomyolysis or cytokine storm may also underline the disease [20]. However, acute tubular injury is considered the most common cause [61]. Ng et al. [61] mention prerenal azotemia, acute tubular injury, glomerular diseases, thrombotic microangiopathy, and treatment-related AKI among the pathological mechanisms of AKI formation in COVID-19. However, Pecly et al. [23] indicate that rhabdomyolysis may also contribute to AKI. The pathomechanism of rhabdomyolysis-induced AKI in the course of COVID-19 is based on the release of significant amounts of myoglobin, which results in myoglobinuria, and this leads to the formation of pigment casts. This results in intratubular obstruction [23].

### 5.2. Treatment of RIAKI in the Course of COVID-19

In the cases described, the therapeutic methods used were individually adapted to the patient and varied. However, a common element was intravenous fluid therapy and oxygen therapy [13,14,15,16,17]. Other therapies used include antiviral therapy [13,14], antibiotic therapy [14,15,16,17], diuretics [13,15], nutritional support [14,15], and electrolyte compensation [13,16]. RRT was necessary in the patients described in the cited case reports who developed AKI [13,15,16,17]. In contrast, Jin et al. [14] emphasize that prompt recognition and initiation of fluid therapy avoided complications of rhabdomyolysis, including AKI. In the case of two of the five patients described, it is reported that they required hospitalization in an ICU [15,16]. Table 5 shows a comparison of case reports describing cases of rhabdomyolysis in COVID-19 [13,14,15,16,17].

## 6. Rhabdomyolysis-Induced AKI (RIAKI) Treatment

The mainstay of rhabdomyolysis treatment is to find and eliminate its cause, and to treat the associated disorders. It is crucial to diagnose and treat potentially fatal electrolyte disorders, such as hypokalemia, hypophosphatemia, hypocalcemia, hypo-/hypernatremia, or hyperglycemic states [27].

The first therapeutic step in patients with rhabdomyolysis is intravenous fluid therapy with balanced crystalloid or 0.9% NaCl in doses up to 1.5 L/h. This is to achieve a diuresis of 300 mL/h [19]. The composition of the fluid used in the above-described therapy remains controversial due to the effects on electrolyte balance. Furthermore, Michelsen et al. [62] recommend the use of crystalloids rather than colloids. Aggressive fluid therapy is particularly important in patients with AKI, as it allows replenishment of the vascular bed, thereby restoring renal perfusion. Increased renal flow reduces the formation of pigment casts, a common complication of rhabdomyolysis [26]. Cabral et al. [26] emphasize that fluid therapy requires close monitoring as it bears the risk of volume overload.

The next step in the treatment of rhabdomyolysis is the administration of sodium bicarbonate to alkalize the urine. The rationale for its use is that heme pigment precipitates in an acidic environment, which increases the toxicity of myoglobin released in large quantities during rhabdomyolysis. It has been shown that elevating urine pH above 6.5 also has the property of reducing the damaging effects of oxidative stress that cause damage to functional and structural components of the kidney [38]. However, the authors of studies emphasize that sodium bicarbonate may cause hypocalcemia [27,38]. Moreover, therapy with this drug is contraindicated in patients with respiratory or circulatory insufficiency due to the risk of hyperosmolar states. The benefits of urinary alkalinization with sodium bicarbonate require further study, especially including a comparison of the described therapy with standard fluid therapy [38].

Another drug used in the treatment of rhabdomyolysis is mannitol. It is a diuretic drug whose mechanism is based on the phenomenon of osmosis [27,38]. Although diuresis leads to an increase in myoglobin excretion [38], both mannitol and furosemide should not be used routinely as there is no evidence to demonstrate the benefits of therapy with these drugs [27]. Moreover, furosemide is known to aggravate urine acidosis [27]. New studies on the pharmacokinetics of furosemide show that its diuretic effect is dependent on the severity of AKI. It has been shown that the effect of furosemide is reduced when diuresis is <500 mL/12 h and/or creatinine clearance is <20 mL/min [63]. Resistance to furosemide and its abnormal metabolism in patients with AKI is due to several factors, which include loss of tubular epithelial polarity, concomitant hypoalbuminemia or impaired urinary excretion of furosemide [64]. In addition, Mariano et al. [63] indicate that a better method of drug delivery is a 20 mg/h continuous infusion. Furosemide administered in this way also allows assessment of glomeruli and tubules by evaluating the effect of the drug thus being an indicator of the presence or absence of mild AKI [63]. The predictive importance of furosemide is also described by McMahon et al. who emphasize the advantages of the furosemide stress test (FST), which are its low cost, safety, and short turnaround time. The FST has found use in spotting patients with an increased probability of AKI progression and predicting its severity. FST also can help to predict the need for RRT and to make the decision to discontinue it. It is also worth noting that in patients with AKI, in the course of COVID-19, hypovolemia is often present. In this case FST is contraindicated until fluid resuscitation is completed [64].

## 7. Renal Replacement Therapy (RRT) in Rhabdomyolysis-Induced AKI (RIAKI)

As CK [65] and myoglobin [41] levels increase, the probability of developing AKI becomes greater [41,65]. It is a condition that may require RRT [26,65]. The need for such treatment is indicated by the occurrence of anuria, elevated serum creatinine levels, and life-threatening electrolyte abnormalities [27]. It is important to implement extracorporeal myoglobin removal in addition to fluid therapy and urine alkalinization [41,66]. Patients with rhabdomyolysis may be treated with continuous renal replacement therapy (CRRT) or daily hemodialysis, whereas peritoneal dialysis is not considered to have the expected therapeutic results [26,27]. In contrast, Guzman et al. [41] reported that hemodialysis is an ineffective method for removing myoglobin and suggested using convective therapy with continuous venous hemofiltration. However, studies have shown that the sieving coefficient decreases in direct proportion to the passage of time, while increasing the flow rate does not significantly increase myoglobin clearance. In addition, a comparison of high cut-off (HCO) dialysis membranes and high-flux membranes revealed better clearance of myoglobin using the first one [41].

**Figure 4:**
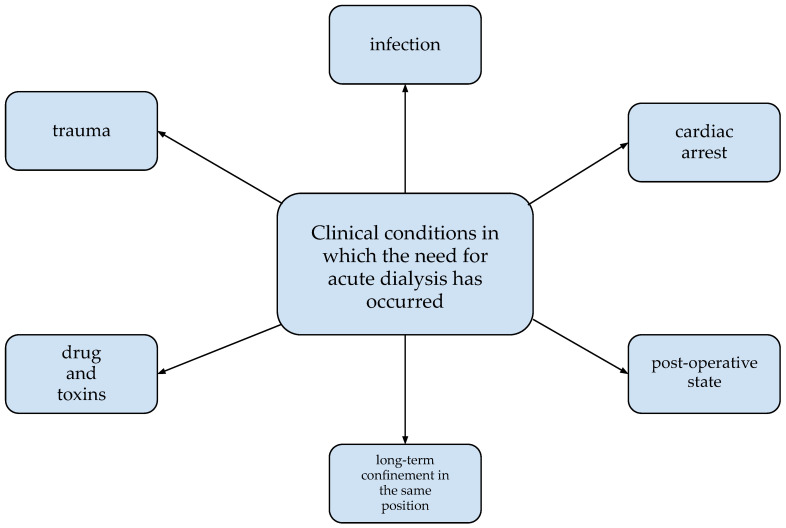
Clinical situations co-occurring with rhabdomyolysis in which the need for dialysis occurred [3].

A variety of factors may contribute to the requirement for RRT in the course of rhabdomyolysis. The most common ones are shown in Figure 4 [3].

The study by Jerman et al. [66] compared the use of HCO and medium cut off (MCO) dialysis membranes and cytokine adsorbers (CytoSorb^®^) in patients with AKI stage 2–3 according to KDIGO and rhabdomyolysis with myoglobin levels >20,000 μg/L. Myoglobin and albumin levels were assessed. Hemodialysis with HCO and MCO results in decreased myoglobin levels. However, it is worth noting that the use of HCO, unlike MCO, causes a significant reduction in albumin levels and makes their substitution necessary. The use of an adsorption capsule carries the benefit of being able to remove both myoglobin and cytokines, which may be applicable, e.g., in multi-organ failure or sepsis with coexisting AKI. Researchers emphasize the usefulness of MCO in RIAKI pointing out the advantages of its use, which are the lack of need for albumin supplementation and lower treatment costs [66].

Scharf et al. [67] emphasize that the removal of myoglobin using conventional dialysis membranes may be difficult due to its molecular weight. They propose the use of a Cytosorb^®^ cytokine adsorber with simultaneous high-flux dialysis, highlighting the efficacy of myoglobin elimination in patients with rhabdomyolysis [67].

## 8. Conclusions

Rhabdomyolysis is a complex disease because it may manifest only as a mild dyselectrolysis while also leading to life-threatening conditions such as AKI. Unfortunately, characteristic symptoms are rare and sometimes non-specific or absent. The diagnostic process is also complicated by the fact that many factors may lead to muscle damage, which may prolong the diagnosis. However, an essential and helpful role in the management is played by the identification of biochemical markers characteristic of damaged muscle tissue. Efficiently performed tests allow to estimate the risk of AKI and to implement appropriate management. Muscle pain, which is commonly associated with COVID-19 disease, in some cases may be a sign of rhabdomyolysis. Therefore, physicians should consider that SARS-CoV-2 can cause rhabdomyolysis and therefore order and perform laboratory tests to diagnose it in certain situations, as early recognition and treatment increase the chances of preventing complications, including but not limited to AKI. Although the most common side effect of the statins is SAMS, recent scientific reports suggest that serious complications such as rhabdomyolysis are rarely observed. Moreover, researchers suggest that in many cases the side effects that occur during statin therapy, including muscle pain, may be due to the nocebo effect, which is the negative attitude study participants have to statins. Treatment of rhabdomyolysis is based primarily on finding and treating the etiology. Aggressive early fluid therapy is considered to be the basis of treatment. In contrast, the use of sodium bicarbonate, mannitol, or furosemide has not demonstrated a clear beneficial effect. AKI, a complication of rhabdomyolysis, may also require RRT, e.g., CRRT or daily hemodialysis.

## Figures and Tables

**Table 1:** KDIGO criteria used to diagnose AKI [24,25,30].

KDIGO Criteria for the Diagnosis of AKI **.	
**1.**	Increase in serum creatinine of at least 0.3 mg/dL in 48 h
**2.**	Increase in serum creatinine by at least 1.5 times in the last 7 days
**3.**	Reduction of the volume of excreted urine below 0.5 mL/kg/h for more than 6 h

** AKI, acute kidney injury.

**Table 2:** Suggested causes of AKI [24,25,29].

The Causes of AKI		
Prerenal	Intrarenal	Postrenal
Hypowolemia	Acute tubular necrosis (prolonged renal ischemia, nephrotoxic agents, sepsis)	Nephrolithiasis/ureteral stones
Hypotension	Acute interstitial nephritis	Tumors in the urinary tract
Renal artery stenosis	Intracellular deposition	Blood clots in the urinary tract
Aortic dissection	Fat embolism	Urethral obstruction
Severe organ failure, e.g., liver failure	Vasculitis	Prostate hypertrophy
Drugs, e.g., NSAIDs, ACEIs, cyclosporine	Rhabdomyolysis	Narrowing of the ureters

NSAIDs, non-steroidal anti-inflammatory drugs; ACEIs, angiotensin converting enzyme inhibitors.

**Table 3:** Examples of causes of muscle damage [2,4,26,35,38].

Acquired	Genetic
Consumption of alcohol and illegal substances	Metabolic myopathies
Prescription medications such as antipsychotics	Channelopathies
Electrolyte and metabolic disturbances	Muscular dystrophies
Pesticide and toxin ingestion	Mitochondrial disorders Disorders of lipid metabolism or glycolysis/glycogenolysis
Injury including crush syndrome Exposure to extreme temperatures e.g., malignant neuroleptic syndrome, heat stroke	
Exertion including intense physical exertion, seizures, epileptic status	
Viral/bacterial infections	

**Table 4:** Suggested SAMS mechanisms [5].

Mechanisms of SAMS Development
The suppression of the synthesis of cholesterol and protein prenylation
Impaired production of high-energy compounds (e.g., ATP *) by mitochondria
Oxidative stress
Apoptosis
The Akt/mTOR pathway impairment

* ATP, adenosine 5′-triphosphate.

**Table 5:** Comparison of patients with SARS-CoV-2-induced rhabdomyolysis [13,14,15,16,17].

Authors	Chedid et al. [13]	Jin et al. [14]	Taxbro et al. [15]	Chetram et al. [16]	Solís et al. [17]
**Patient characteristics**	51-year-old man	60-year-old man	38-year-old man	62 year-old man	46-year-old man
**Comorbidities**	- hypertension - DM type 2 - OSA - CKD stage 2	no information	- DM type 2 - gout - mild obesity	- hypertension - obesity (BMI of 39.6) - DM type 2	- chronic myeloid leukemia
**Symptoms**	- diffuse myalgia (involving chest, back, arms and legs) - dry cough - mild chills	- cough - fever up to 38.3 °C - pain and weakness in the lower extremities (on the 9th day of hospitalization)	- fever - myalgia - nausea and vomiting - dry cough - breathlessness - abdominal pain	- general malaise - poor appetite - decreased urine output - haematuria - fever up to 38.1 °C	- cough - fever - dyspnea - generalized muscle pain
**Laboratory abnormalities (peak levels):**					
Creatinine	19.09 mg/dL	74.4 µmol/L	278 µmol/L	>9, <10 mg/dL (value read from the graph)	16.25 mg/dL
LDH	>2150 U/L	2347 U/L	8.9 μkat/L	no information	26,967 U/L
CK	464,000 U/L	17,434 U/L	no information	327,629 U/L	426,700 U/L
Myoglobin	15,175 mg/L	12,550 µg/L	>21,000 μg/L	no information	no information
cTnT	708 ng/L	no information	444 ng/L	no information	no information
AST	1173 U/L	373 U/L	not reported	1077 U/L (no information if this is the peak value)	3565 U/L
**Applied Renal Replacement Therapy**	intermittent hemodialysis	-	CRRT-continuous venovenous haemodiafiltration	CRRT	CRRT- continuous veno-venous hemodiafiltration
**Course/duration of hospitalization**	discharged on day 15	no information	discharged after 23 days	discharged on day 26	died during hospitalization

DM, diabetes mellitus; OSA, obstructive sleep apnea; CKD, chronic kidney disease; CK, serum creatine kinase; LDH, lactate dehydrogenase; cTnT, troponin T; AST, aspartate aminotransferase; CRRT, continuous renal replacement therapy.

## Data Availability

The data used in this article are sourced from materials mentioned in the References section.

## References

[1] Stahl K., Rastelli E., Schoser B. (2020). A systematic review on the definition of rhabdomyolysis. J. Neurol..

[2] Chavez L.O., Leon M., Einav S., Varon J. (2016). Beyond muscle destruction: A systematic review of rhabdomyolysis for clinical practice. Crit. Care.

[3] McKenna M.C., Kelly M., Boran G., Lavin P. (2019). Spectrum of rhabdomyolysis in an acute hospital. Ir. J. Med. Sci..

[4] Ms J.R.N., Mammen A.L. (2015). Diagnostic evaluation of rhabdomyolysis. Muscle Nerve.

[5] Bouitbir J., Sanvee G.M., Panajatovic M.V., Singh F., Krähenbühl S. (2020). Mechanisms of statin-associated skeletal muscle-associated symptoms. Pharmacol. Res..

[6] Mach F., Baigent C., Catapano A.L., Koskinas K.C., Casula M., Badimon L., Chapman M.J., De Backer G.G., Delgado V., Ference B.A. (2019). 2019 ESC/EAS Guidelines for the management of dyslipidaemias: Lipid modification to reduce cardiovascular risk. Eur. Heart J..

[7] Singh B.M., Lamichhane H.K., Srivatsa S.S., Adhikari P., Kshetri B.J., Khatiwada S., Shrestha D.B. (2020). Role of Statins in the Primary Prevention of Atherosclerotic Cardiovascular Disease and Mortality in the Population with Mean Cholesterol in the Near-Optimal to Borderline High Range: A Systematic Review and Meta-Analysis. Adv. Prev. Med..

[8] Thompson P.D., Panza G., Zaleski A., Taylor B. (2016). Statin-Associated Side Effects. J. Am. Coll. Cardiol..

[9] Baigent C., Blackwell L., Emberson J., Holland L.E., Reith C., Bhala N., Collins R., Cholesterol Treatment Trialists’ (CTT) Collaboration (2010). Efficacy and safety of more intensive lowering of LDL cholesterol: A meta-analysis of data from 170 000 participants in 26 randomised trials. Lancet.

[10] Grundy S.M., Stone N.J., Bailey A.L., Beam C., Birtcher K.K., Blumenthal R.S., Braun L.T., de Ferranti S., Faiella-Tommasino J., Forman D.E. (2018). 2018 AHA/ACC/AACVPR/AAPA/ABC/ACPM/ADA/AGS/APhA/ASPC/NLA/PCNA Guideline on the Management of Blood Cholesterol: Executive Summary: A Report of the American College of Cardiology/American Heart Association Task Force on Clinical Practice Guidelines. J. Am. Coll. Cardiol..

[11] Guan W.J., Ni Z.Y., Hu Y., Liang W.H., Qu C.Q., He J.X., Liu L., Shan H., Lei C.L., Hui D.S.C. (2020). China medical treatment expert group for COVID-19 2020. Clinical Characteristics of coronavirus disease in China. N. Engl. J. Med..

[12] World Health Organization COVID-19 Weekly Epidemiological Update Edition 93, Published 25 May 2022. https://www.who.int/publications/m/item/weekly-epidemiological-update-on-covid-19.

[13] Chedid N.R., Udit S., Solhjou Z., Patanwala M.Y., Sheridan A.M., Barkoudah E. (2020). COVID-19 and Rhabdomyolysis. J. Gen. Intern. Med..

[14] Jin M., Tong Q. (2020). Rhabdomyolysis as Potential Late Complication Associated with COVID-19. Emerg. Infect. Dis..

[15] Taxbro K., Kahlow H., Wulcan H., Fornarve A. (2020). Rhabdomyolysis and acute kidney injury in severe COVID-19 infection. BMJ Case Rep..

[16] Chetram V.K., Ahmad A.I., Farid S., Sood T. (2021). Acute Kidney Injury Secondary to Rhabdomyolysis and COVID-19: A Case Report and Literature Review. Case Rep. Nephrol..

[17] Solís J.G., Pineda A.E., Minutti P.A., Sánchez A.A. (2020). Case Report: Rhabdomyolysis in a Patient with COVID-19: A Proposed Diagnostic-Therapeutic Algorithm. Am. J. Trop. Med. Hyg..

[18] Ramos-Casals M., Brito-Zerón P., Mariette X. (2021). Systemic and organ-specific immune-related manifestations of COVID-19. Nat. Rev. Rheumatol..

[19] Hannah J.R., Ali S.S., Nagra D., Adas M.A., Buazon A.D., Galloway J.B., Gordon P.A. (2022). Skeletal muscles and COVID-19: A systematic review of rhabdomyolysis and myositis in SARS-CoV-2 infection. Clin. Exp. Rheumatol..

[20] Rosen H.R., O’Connell C., Nadim M.K., DeClerck B., Sheibani S., DePasquale E., Sanossian N., Blodget E., Angell T. (2021). Extrapulmonary manifestations of severe acute respiratory syndrome coronavirus-2 infection. J. Med Virol..

[21] Sabaghian T., Kharazmi A.B., Ansari A., Omidi F., Kazemi S.N., Hajikhani B., Vaziri-Harami R., Tajbakhsh A., Omidi S., Haddadi S. (2022). COVID-19 and Acute Kidney Injury: A Systematic Review. Front. Med..

[22] Sharma P., Uppal N.N., Wanchoo R., Shah H.H., Yang Y., Parikh R., Khanin Y., Madireddy V., Larsen C.P., Jhaveri K.D. (2020). COVID-19–Associated Kidney Injury: A Case Series of Kidney Biopsy Findings. J. Am. Soc. Nephrol..

[23] Pecly I.M.D., Azevedo R.B., Muxfeldt E.S., Botelho B.G., Albuquerque G.G., Diniz P.H.P., Silva R., Rodrigues C.I.S. (2021). A review of COVID-19 and acute kidney injury: From pathophysiology to clinical results. J. Bras. Nefrol..

[24] Goyal A., Daneshpajouhnejad P., Hashmi M.F., Bashir K. (2022). Acute Kidney Injury.

[25] Goyal A., Daneshpajouhnejad P., Hashmi M.F., Bashir K., John B.K. (2022). Acute Kidney Injury (Nursing).

[26] Cabral B.M.I., Edding S.N., Portocarrero J.P., Lerma E.V. (2020). Rhabdomyolysis. Disease-A-Month.

[27] Long B., Koyfman A., Gottlieb M. (2019). An evidence-based narrative review of the emergency department evaluation and management of rhabdomyolysis. Am. J. Emerg. Med..

[28] National Clinical Guideline Centre (UK) (2013). Acute Kidney Injury: Prevention, Detection and Management Up to the Point of Renal Replacement Therapy.

[29] Bindroo S., Quintanilla Rodriguez B.S., Challa H.J. (2022). Renal Failure.

[30] National Institute for Health and Care Excellence (NICE) (2019). Acute Kidney Injury: Prevention, Detection and Management.

[31] Hanif M.O., Bali A., Ramphul K. (2022). Acute Renal Tubular Necrosis.

[32] Manzoor H., Bhatt H. (2022). Prerenal Kidney Failure.

[33] Naik R.H., Annamaraju P. (2022). Interstitial Nephritis.

[34] Naik R.H., Shawar S.H. (2022). Rapidly Progressive Glomerulonephritis.

[35] Bosch X., Poch E., Grau J.M. (2009). Rhabdomyolysis and Acute Kidney Injury. N. Engl. J. Med..

[36] Thakore P., Liang T.H. (2022). Urolithiasis.

[37] Bajema I.M., Rotmans J.I. (2018). Histological manifestations of rhabdomyolysis in the kidney. Nephrol. Dial. Transplant..

[38] Somagutta M.R., Pagad S., Sridharan S., Nanthakumaran S., Arnold A.A., May V., Malik B.H. (2020). Role of Bicarbonates and Mannitol in Rhabdomyolysis: A Comprehensive Review. Cureus.

[39] Yang J., Zhou J., Wang X., Wang S., Tang Y., Yang L. (2020). Risk factors for severe acute kidney injury among patients with rhabdomyolysis. BMC Nephrol..

[40] Weidhase L., De Fallois J., Haußig E., Kaiser T., Mende M., Petros S. (2020). Myoglobin clearance with continuous veno-venous hemodialysis using high cutoff dialyzer versus continuous veno-venous hemodiafiltration using high-flux dialyzer: A prospective randomized controlled trial. Crit. Care.

[41] Guzman N., Podoll A.S., Bell C.S., Finkel K.W. (2013). Myoglobin Removal Using High-Volume High-Flux Hemofiltration in Patients with Oliguric Acute Kidney Injury. Blood Purif..

[42] Simpson J.P., Taylor A., Sudhan N., Menon D.K., Lavinio A. (2016). Rhabdomyolysis and acute kidney injury: Creatine kinase as a prognostic marker and validation of the McMahon Score in a 10-year cohort: Retrospective observational evaluation. Eur. J. Anaesthesiol..

[43] Vangstad M., Bjornaas M.A., Jacobsen D. (2019). Rhabdomyolysis: A 10-year retrospective study of patients treated in a medical department. Eur. J. Emerg. Med..

[44] Fodili F., Van Bommel E.F.H. (2003). Severe rhabdomyolysis and acute renal failure following recent Coxsackie B virus infection. Neth. J. Med..

[45] Chandel A., Brusher K., Hall V., Howard R.S., Clark P.A. (2019). Diagnosis and Management of Rhabdomyolysis in the Absence of Creatine Phosphokinase: A Medical Record Review. Mil. Med..

[46] Rebalka I.A., Hawke T.J. (2014). Potential biomarkers of skeletal muscle damage. Biomark. Med..

[47] Banfi G., Colombini A., Lombardi G., Lubkowska A. (2012). Metabolic markers in sports medicine. Adv Clin Chem..

[48] Brancaccio P., Lippi G., Maffulli N. (2010). Biochemical markers of muscular damage. Clin. Chem. Lab. Med. (CCLM).

[49] Beigvand H.H., Heidari K., Hashemi B., Saberinia A. (2021). The Value of Lactate Dehydrogenase in Predicting Rhabdomyolysis-Induced Acute Renal Failure; a Narrative Review. Arch. Acad. Emerg. Med..

[50] Lavallaz J.D.F.D., Zehntner T., Puelacher C., Walter J., Strebel I., Rentsch K., Boeddinghaus J., Nestelberger T., Twerenbold R., Mueller C. (2018). Rhabdomyolysis. J. Am. Coll. Cardiol..

[51] Ayala E., Kagawa F.T., Wehner J.H., Tam J., Upadhyay D. (2009). Rhabdomyolysis Associated With 2009 Influenza A(H1N1). JAMA.

[52] Istvan E.S., Deisenhofer J. (2001). Structural Mechanism for Statin Inhibition of HMG-CoA Reductase. Science.

[53] Kajinami K., Tsukamoto K., Koba S., Inoue I., Yamakawa M., Suzuki S., Hamano T., Saito H., Saito Y., Masuda S. (2020). Statin Intolerance Clinical Guide 2018. J. Atheroscler. Thromb..

[54] Newman C.B., Preiss D., Tobert J.A., Jacobson T.A., Page I.R.L., Goldstein L.B., Chin C., Tannock L.R., Miller M., Raghuveer G. (2019). Statin Safety and Associated Adverse Events: A Scientific Statement From the American Heart Association. Arter. Thromb. Vasc. Biol..

[55] Coste J., Billionnet C., Rudnichi A., Pouchot J., Dray-Spira R., Giral P., Zureik M. (2019). Statins for primary prevention and rhabdomyolysis: A nationwide cohort study in France. Eur. J. Prev. Cardiol..

[56] Finegold J.A., Manisty C., Goldacre B., Barron A.J., Francis D.P. (2014). What proportion of symptomatic side effects in patients taking statins are genuinely caused by the drug? Systematic review of randomized placebo-controlled trials to aid individual patient choice. Eur. J. Prev. Cardiol..

[57] Gupta A., Thompson D., Whitehouse A., Collier T., Dahlof B., Poulter N., Collins R., Sever P. (2017). Adverse events associated with unblinded, but not with blinded, statin therapy in the Anglo-Scandinavian Cardiac Outcomes Trial—Lipid-Lowering Arm (ASCOT-LLA): A randomised double-blind placebo-controlled trial and its non-randomised non-blind extension phase. Lancet.

[58] Chatzizisis Y.S., Misirli G., Hatzitolios A.I., Giannoglou G.D. (2008). The syndrome of rhabdomyolysis: Complications and treatment. Eur. J. Intern. Med..

[59] Candela N., Silva S., Georges B., Cartery C., Robert T., Moussi-Frances J., Rondeau E., Rebibou J.-M., Lavayssiere L., Belliere J. (2020). Short- and long-term renal outcomes following severe rhabdomyolysis: A French multicenter retrospective study of 387 patients. Ann. Intensiv. Care.

[60] Noble R.A., Selby N.M. (2022). The changing nature of COVID-19-associated AKI: Where are we now?. Nephrol. Dial. Transplant..

[61] Ng J.H., Bijol V., Sparks M.A., Sise M.E., Izzedine H., Jhaveri K.D. (2020). Pathophysiology and Pathology of Acute Kidney Injury in Patients With COVID-19. Adv. Chronic Kidney Dis..

[62] Michelsen J., Cordtz J., Liboriussen L., Behzadi M.T., Ibsen M., Damholt M.B., Møller M.H., Wiis J. (2019). Prevention of rhabdomyolysis-induced acute kidney injury–A DASAIM/DSIT clinical practice guideline. Acta Anaesthesiol. Scand..

[63] Mariano F., Mella A., Vincenti M., Biancone L. (2019). Furosemide as a functional marker of acute kidney injury in ICU patients: A new role for an old drug. J. Nephrol..

[64] McMahon B.A., Chawla L.S. (2021). The furosemide stress test: Current use and future potential. Ren. Fail..

[65] Nielsen F.E., Cordtz J.J., Rasmussen T.B., Christiansen C.F. (2020). The Association Between Rhabdomyolysis, Acute Kidney Injury, Renal Replacement Therapy, and Mortality. Clin. Epidemiol..

[66] Jerman A., Andonova M., Persic V., Gubensek J. (2022). Extracorporeal Removal of Myoglobin in Patients with Rhabdomyolysis and Acute Kidney Injury: Comparison of High and Medium Cut-Off Membrane and an Adsorber Cartridge. Blood Purif..

[67] Scharf C., Liebchen U., Paal M., Irlbeck M., Zoller M., Schroeder I. (2021). Blood purification with a cytokine adsorber for the elimination of myoglobin in critically ill patients with severe rhabdomyolysis. Crit. Care.

